# Obtaining elevation of *Oncomelania Hupensis* habitat based on Google Earth and it’s accuracy evaluation: an example from the Poyang lake region, China

**DOI:** 10.1038/s41598-020-57458-0

**Published:** 2020-01-16

**Authors:** Fei Hu, Jun Ge, Chunfang Lu, Qiyue Li, Shangbiao Lv, Yifeng Li, Zhaojun Li, Min Yuan, Zhe Chen, Yueming Liu, Ying Liu, Dandan Lin

**Affiliations:** 1 Jiangxi Provincial Institute of Parasitic Diseases, Nanchang, 330096 China; 2 Jiangxi Province Key Laboratory of Schistosomiasis Prevention and Control, Nanchang, 330096 China; 3 Jiangxi Normal University, Nanchang, 330022 China

**Keywords:** Environmental sciences, Space physics, Risk factors

## Abstract

*Schistosomiasis japonicum* is a major zoonosis that seriously harms human health and affects social and economic development in China. The control of *Oncomelania Hupensis*, the only intermediate host of *schistosome japonicum*, is one of the integrated measures for schistosomiasis control in China. Acquiring updated elevation data of snail habitat environment, as well as it’s spatial analysis, play an important role for the risk evaluation and precise control of schistosomiasis transmission and prevalence. Currently, the elevation database of snail habitat environment in schistosomiasis epidemic areas has not been available in the world, which affects the development of research and application work regarding to snail control. Google Earth(GE) can provide massive information related to topography, geomorphology and ground objects of a region due to its indisputable advantages such as wide use, free charge and rapidly updating. In this paper, taking the Poyang lake region as a example, we extracted elevation data of snail-inhabited environment of the lake from GE and established a elevation correction regression model(CRM) for acquiring accurate geospatial elevations, so as to provide a decision-making reference for snail control and risk evaluation of schistosomiasis in China. We developed a GE Application Programming Interface(API) program to extract elevation data from GE, which was compared with the actual elevation data obtained from topographic map of the Poyang Lake bottom. Then, a correction regression model was established and evaluated by 3 index, Mean Absolute Error(MAE), Root Mean Squared Error(RMSE) and Index of Agreement(IOA) for the accuracy of the model. The elevation values extracted from GE in 15086 sample grid points of the lake ranged from 8.5 m to 24.8 m. After the sample points were divided randomly to three groups, the mean elevations of three groups were 13.49 m, 13.52 m and 13.65 m, respectively, with standard deviation ranged from 2.04–2.06. The mean elevation among three groups has no statistic difference (F = 1.536, P = 0.215). A elevation correction regression model was established as y = 6.228 + 0.485×. the evaluation results for the accuracy of the model showed that the MAE and RMSE before correction was 1.28 m and 3.95 m respectively, higher than that after correction, which were 0.74 and 1.30 m correspondingly. The IOA before correction (−0.40)was lower than that after correction(0.34). Google Earth can directly or indirectly get access to massive information related to topography, geomorphology and ground objects due to its indisputable advantages. However, it still needs to be converted into more reliable and accurate data by combining with pre-processing tools. This study used self-developed API program to extract elevation data from GE through precisely locating and improved the accuracy of elevation by using a correction regression model, which can provide reliable data sources for all kinds of spatial data researches and applications.

## Introduction

*Schistosomiasis japonicum* is one of the major zoonosis that seriously harms human health and affects social and economic development and remains a public health problem in China’s southern 12 provinces^1,2^. The breeding and development of *Oncomelania hupensis*, the only intermediate host of *Schistosoma japonicum*, require specific spatial environment due to it’s biological characteristics. The distribution of snail is closely related to it’s living environment including climate, elevation, soil, water level and vegetation^3^. Previous studies have shown that the elevation of snail-inhabited environment is relatively stable in local region such as the Poyang Lake^4,5^. However, the elevation with frequent snail activity and the snail distribution range vary with the increase of space distance. In recent years, a trend of snail-inhabited environment moving down to low elevation has been showed in some parts of endemic areas with schistosomiasis in China^6–8^. What’s more notable is that, after entered 2000s, under the integrated influence of natural and anthropogenic factors such as the regional climate change, Three Gorges Dam operation, and sand mining, the water level of Poyang Lake showed a continuous decreasing trend. The shrinking size, advanced and prolonged dry season, which will definitely have an significant impact on the snails breeding environment^9–12^. Therefore, timely obtaining the change information of elevation, size and scope of snail-inhabited environment is crucial for scientifically formulating mollusciciding and rationally controlling schistosomiasis.

Digital Elevation Model(DEM) is an important spatial information dataset in the basic Geographic Information System database, and is also the core component for spatial terrain analysis. DEM has been widely used in various fields including schistosomiasis control due to the diversity of its data forms^13–15^. The traditional large-scale ground surveying and mapping method has high accuracy, while it has high cost and low timeliness and effectiveness when it used on a large scale. Moreover, it still lacks of applications for large-scale and high-resolution mapping of snail-inhabited environments specifically. Compared with the traditional large-scale ground surveying and mapping, high-resolution remote sensing and radar technology can quickly and efficiently obtain the relevant information of snail-inhabited environment, but cannot obtain accurate elevation data of these areas^16,17^.

Google Earth(GE) is a virtual globe software developed by Google company, which has huge amount of submeter-level resolution satellite image data, and keep update. GE offered high-resolution elevation data, which started in June 2005 and used Shuttle Radar Topography Mission (SRTM) data for its elevation baseline, it gains wide recognition for it’s remarkably improving the visualization and dissemination of scientific data. In recent years, some visualization techniques based on GE have been widely used in schistosomiasis control and research^8,18–22^. In addition, GE shares latitude, longitude and elevation information simultaneously, and provides Application Programming Interface(API) that allows users to develop API program to extract elevation data from anywhere around the world. The elevation extracted from GE has higher accuracy and resolution, and plays an important role in many practical applications^23–25^. Moreover, the elevation value shown by GE is based on World Geodetic System-1984 Coordinate System(WGS-84), rather than the 1985 Yellow Sea elevation system, which is commonly used in China. The elevation data from these two system can not be inter-converted.

In this study, we developed a GE API program (DEM Tools Pro, 2015) to obtain elevation data of snail-inhabited environment digitally by loading the high resolution remote sensing images of GE. Meanwhile, for the first time, we compared the GE elevation with the actual elevation obtained from the topographic map of the bottom of Poyang Lake for accuracy analysis and established a correction regression model(conversion model). Taking the largest fresh water lake in China for example, this paper obtained elevation data of snail-inhabited environment by using GE and established a correction regression model for accurate elevation data, attempting to provide reference for mollusciciding and schistosomiasis control around the world.

## Methods

### Study area

**Figure 1:**
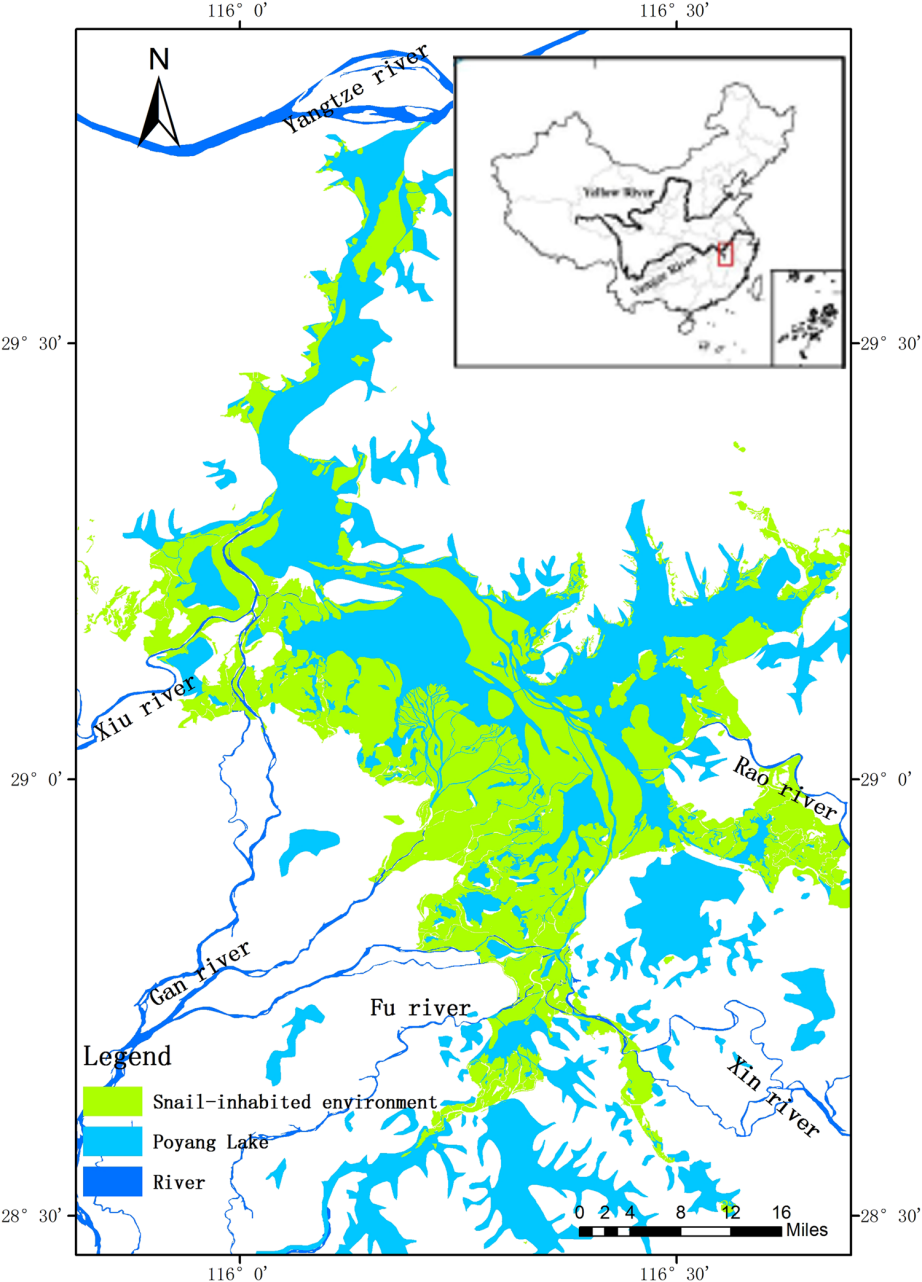
Map of the geospatial location of the Poyang Lake overlaid by China administrative vectorgraph, Poyang lake vectorgraph, snail-inhabited environment vectorgraph by ArcGIS 10.2, https://support.esri.com/zh-cn/products/desktop/arcgis-desktop/arcmap/10-2-2.

The Poyang Lake, the largest freshwater lake in China, was selected for this study. Located in the northern part of Jiangxi Province(28°11′∼29°51′N 115°4, 9′∼116°46′E), the Poyang Lake is a water-carrying and influent-effluent lake naturally connected with the Yangtze River (Fig. 1). The five major rivers and their tributaries run through the whole Jiangxi province and all flow into the Poyang Lake. In dry seasons (Spring and Winter), the marshlands of the lake emerged from water with thriving vegetation. Contrarily, in flood seasons(Summer and Autumn), the marshlands were submerged under water^26^. It has showed from previous studies that the water level of the lake changed between 7.1–19.0 m (1985 National Yellow Sea Elevation Benchmarks). The historical data showed that the water level changed between 7.1–19.0 m in one year^27^. The ecological factors in the lake, including climate, geographical environment, hydrological characteristics, vegetation, activities of human and cattle, were contributed to the breeding and development of *Oncomelania hupensis* and the transmission of schistosomiasis, giving rise to that the Poyang Lake region has long been the most serious schistosomiasis epidemic areas in Jiangxi Province.

### Sampling grids

The map of the snail-inhabited marshlands was established using ArcGIS with WGS-84 geographic coordinate system^28^ and Transverse Mercator projection system. Total 62340 vector grids by 200 × 200 m were created on the map of marshlands through Hawth’s Tools in ArcGIS, of which, 37754 grids were selected as valid grids when its actual area was above 24000 m^2^. Of 37754 valid grids, 15086 grids were randomly selected as sample grids for this study by using Create Random Selection tool in ArcGIS. A field survey was conducted in 2016. Each sample grid was surveyed for snails with taking one side of each grid as the stating line. In each grid one survey frame of 0.1 m^2^ was set up with in 50 m × 50 m area. There were 25 spots in each grid that were surveyed and the GPS coordinates of each spot were recorded^29^.

### Obtainment of elevation data

#### DEM elevation of poyang lake

First, 241 topographic maps of the lake bottom with a scale of 1:10000 were scanned into digital images, and using Geoway software, these digital images were transferred to vector data with property code. Then, the elevation spots with value less than 22 m were extracted from the topographic vector data and were processed to obtain a topographic elevation dataset of the lake bottom through procedures including data format conversion, coordinate system transformation, data editing, data splicing, etc. Finally, using ArcGIS 3D spatial analysis tool, the DEM of the lake bottom was generated from the elevation dataset^30^, and the elevation of each sample spot was extracted from the generated DEM (DEM elevation).

#### Google earth elevation

GE provides elevation data that can be obtained through GE Application Programming Interface (API)^15^. The elevation of the same sample spots can be extracted directly based on the spot coordinates by using GE API. We use Delphi language to develop the DEM Tools Pro program to extract elevation of sample spots from GE(GE elevation).

### Evaluation methods

#### Comparison between DEM elevation and GE elevation

The sample spots were randomly divided into three groups, and the absolute error values of DEM elevation and GE elevation of each sample spot were calculated. The descriptive statistical analysis method with 3 indexes, mean value, standard deviation and coefficient variation, was compared whether there were differences in error value among 3 groups, in order to evaluate the reliability and stability of GE elevation.

#### Establishment of correction regression model of elevation (CRM)

**Figure 2:**
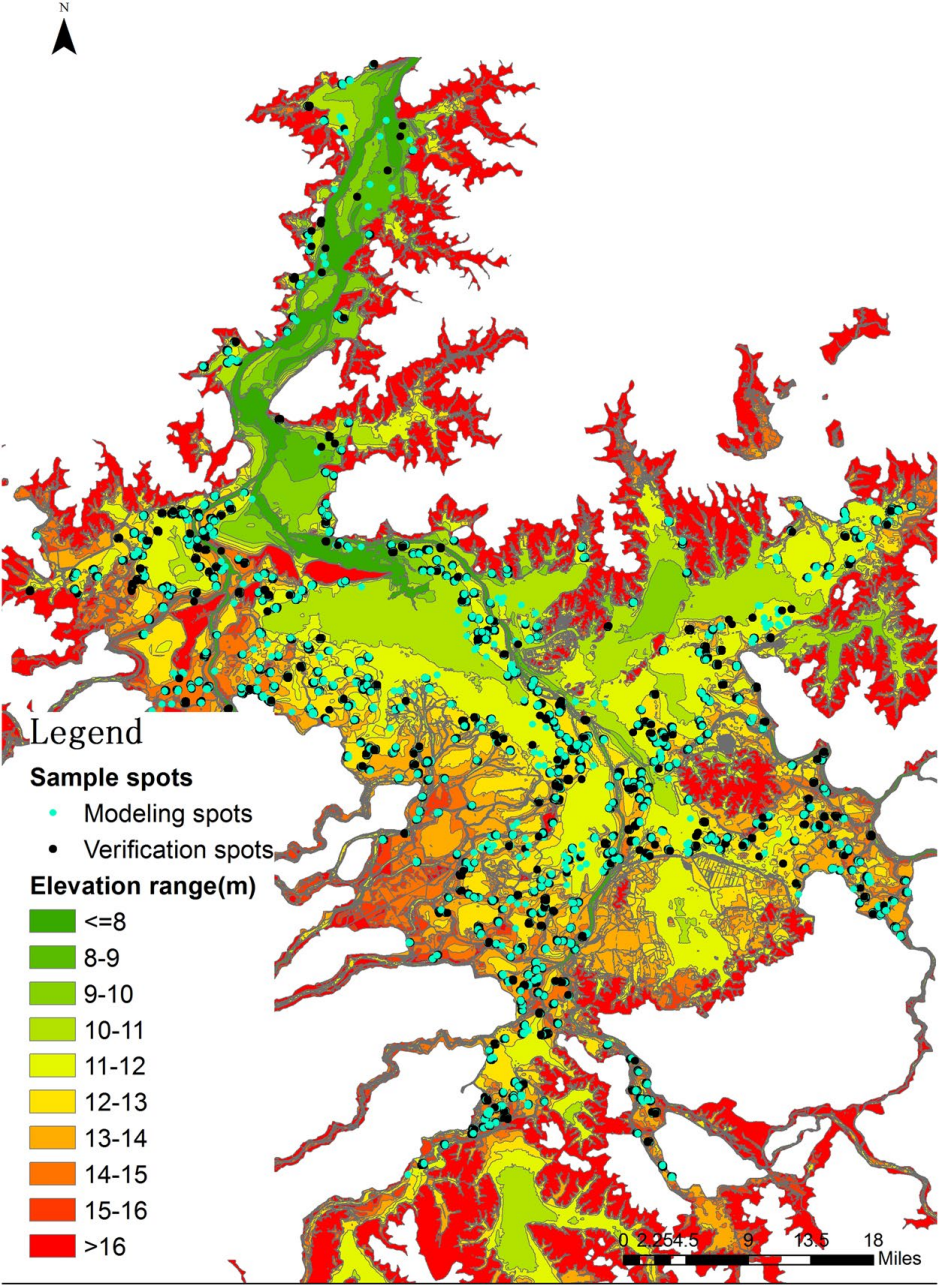
Geo-spatial distribution map of different elevation sample spots in the Poyang Lake region overlaid by sample spots coordinate and Poyang lake bottom terrain vetorgraph by ArcGIS 10.2, https://support.esri.com/zh-cn/products/desktop/arcgis-desktop/arcmap/10-2-2.

70% of sample spots, from which GE elevation and DEM elevation had extracted, were randomly selected for establishing Correction Regression Model of elevation, and other 30% of sample spots were used to evaluate the accuracy of the model (Fig. 2). The relationship between GE elevation and DEM elevation was analyzed by Pearson correlation. The elevation regression model used linear regression equation determined by least square method to correct GE elevation. The model is expressed as following regression equation: y = a + bx, where y represents the dependent variable, a and b represent the constant value and the regression coefficient, respectively, and x represent the independent variable. In this study, the GE elevation value is the independent variable with the corrected elevation value being dependent variable, and R^2^ is the fitting degree of the equation. The closer the R^2^ value is to 1, the better the fitting degree is, and vice versa.

### Accuracy evaluation of elevation

Three indexes, Mean Absolute Error (MAE), Root Mean Squared Error (RMSE and Index of Agreement (IOA), were used to evaluate the accuracy of the elevation Correction Regression Model. MAE reflects the possible error range of the predicted value. RMSE reflects the inversion sensitivity and extreme value effect of the model. The smaller the value of MAE and RMSE, the higher the data accuracy of the model. IOA reflects the fitting degree of the model. The larger the value, the better the fitting effect. We used the GE elevation data of 4533 sample spots to calculate MAE, RMSE and IOA before and after the correction by the Correction Regression Model, respectively. The GE elevations before and after correction were compared with the actual elevation of DEM for evaluating the accuracy of the model. The expression equations for each index were listed as follows, where *P*_*i*_ represents the actual value of the elevation of the i verification sample point, ${P}_{i}^{\ast }$ represents the predicted GE elevation value of the i verification sample point, *n* represents the number of verification sample points, $\overline{{P}_{i}}$ represents the average of the actual elevation of n verification sample points, and P represents the average of the predicted GE elevation value of n verification sample points.

$$MAE=\frac{{\sum }_{i=1}^{n}|{P}_{i}-{P}_{i}^{\ast }|}{n}$$

$$RMSE=\sqrt{\frac{{\sum }_{i=1}^{n}{({P}_{i}-{P}_{i}^{\ast })}^{2}}{n}}$$

$$IOA=1-|\frac{{\sum }_{i=1}^{n}{({P}_{i}-{P}_{i}^{\ast })}^{2}}{{\sum }_{i=1}^{n}(|{P}_{i}-\overline{{P}_{i}}|+|{P}_{i}^{\ast }-\overline{{P}_{i}^{\ast }}|)}|$$

## Results

### Descriptive statistical analysis

The mean values of DEM elevation in three groups were 12.77 m, 12.77 m and 12.82 m, respectively, with no statistical difference among groups (t = 1.924, P = 0.146), and the mean values of GE elevation in three groups were 13.53 m, 13.57 m and 13.60 m, respectively, without statistical difference among groups (t = 1.565, P = 0.209). All mean values of GE elevation in three groups were significant higher than those of DEM elevation (t_1_ = −37.574, t_2_ = −39.284, t_3_ = −40.092, P_*All*_ = 0.000). the mean absolute error between DEM elevation and GE elevation in three groups was 1.47 m, 1.48 m and 1.46, respectively, and the standard deviation ranged from 1.10–1.12 m in three groups. Both mean absolute error and standard deviation showed no different significantly among groups (t = 0.543, P = 0.581). CV varied negligible among groups, ranging from 73.87–75.68% (Table 1).

**Table 1:** The values of DEM elevation and GE elevation in three groups in Poyang Lake.

Group	No. of sample spots	Average Elevation (m)		Absolute Error (m)		
		DEM	GE	Mean	SD	CV(%)
1	5029	12.77	13.53	1.47	1.11	75.51
2	5024	12.77	13.57	1.48	1.12	75.68
3	5033	12.82	13.60	1.46	1.08	73.97
Total	15086	12.79	13.57	1.47	1.10	74.83

Both mean DEM elevation of CRM-establishing samples and CRM-validating samples in Poyang Lake were 12.79 m. The coefficient of variation (CV) between these two groups had no significant difference, ranging from 10.87 to 11.10%, which indicate that there was no significant difference between the mean elevation of CRM-establishing samples and CRM-validating samples (Table 2).

**Table 2:** Comparison between DEM mean elevation of samples for establishing CRM and for validating CRM in Poyang Lake.

Group of samples	No. of samples	Min. (m)	Max. (m)	Mean (m)	SD	CV(%)
Total	15086	5.0	19.7	12.79	1.39	10.87
CRM-establishing samples	10533	5.5	19.1	12.79	1.42	11.10
CRM-validating samples	4533	5.0	19.7	12.79	1.40	10.95

### Elevation correction regression model

There was a significant positive correlation between GE elevation and DEM elevation in Poyang Lake by using Pearson Correlation Analysis (R = 0.917, p = 0.000). The elevation correction regression equation was *y* = 6.228 + 0.485*x* (R^2^ = 0.841, t = 11505.845, P = 0.000, DW = 1.979). Both the intercept and the slope were passed t test (t_*a*_ = 107.265, t_*b*_ = 100.656, P_*all*_ = 0.000), meaning a better the fitting degree.

### Accuracy evaluation of the model

**Figure 3:**
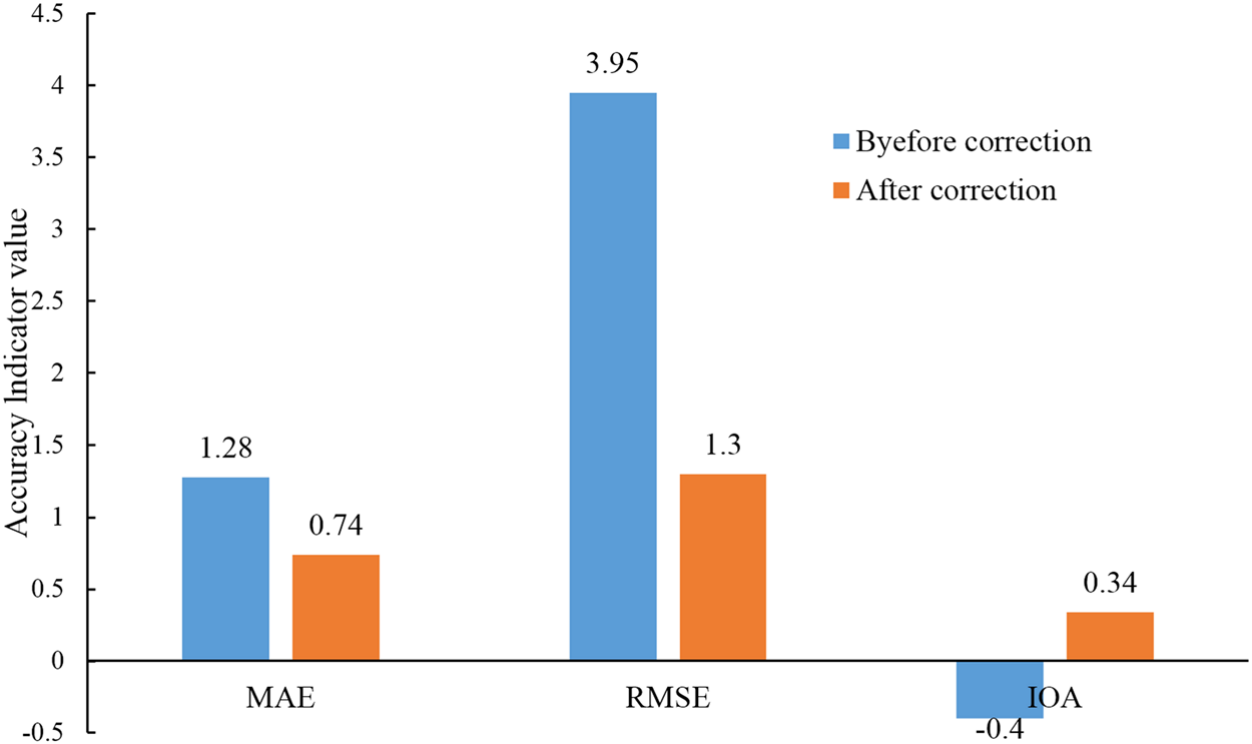
Indicators of MAE, RMS and IOA of GE elevation before and after correction for accuracy evaluation of the model.

MAE and RMSE were decreased from 1.28 m and 3.95 m before correction to 0.74 m and 1.30 m after correction, respectively. IOA before correction was −0.40, lower than 0.34 after correction (Fig. 3). It was indicated that the accuracy of the corrected GE elevation is higher than that obtained directly from GE when both compared with the DEM elevation.

## Discussion

Elevation data are essential for spatial analysis in many fields including schistosomiasis control. However, high-accuracy elevation data are not always available in many areas for researchers and practitioners. Some researchers have used GE elevation data in their scope of researches^8,15,31–34^. However GE images are not orthographic and do not have photogrammetric accuracy. They are collected from satellite images with world-wide coverage, hence, do not have an identical spatial resolution or positional accuracy over the globe^35^. Therefore, it is strongly suggested by^36^ that the users of Google Earth have to test the accuracy of elevation data by comparing with reference data before using it. Hoffmann E. *et al*.^23^ examined that Google Earth TM,s elevation data were at a resolution 5 to 20 times higher than available South African 1:50000 Chief Directorate Surveys and Mapping (CDSM) datasets. Nevertheless, in some areas the root mean square error of NASA’s Shuttle Radar Topography Mission (SRTM) DEM was more than its specified accuracy of ±16m^37^. HQ *et al*.^38^ obtained the elevation data obtained from GE and Shuttle Radar Topography Mission(SRTM3) DEM, respectively. Compared with the actual elevation, both obtained elevation by these two methods were higher. Sharma A. *et al*.^37^ assessed the horizontal positional accuracy of GE in Montreal, the result revealed that the positional accuracy varies between ~0.1 m in the south to ~2.7 m in the north. In this paper, taking the Poyang Lake as a example, we attempted to extract elevation data from GE, compared the data with that from the lake DEM, and established a more accurate elevation conversion model based on GE to fill the gap.

In this paper, 15,806 sample spots in the Poyang lake region were randomly divided into 3 groups to compare the mean elevation values extracted from GE and DEM(actual elevation). The results revealed that the GE elevation values were higher than that of DEM in three groups, meaning there may exist errors between them. The results also illustrated that the mean elevation absolute error ranged from 1.46–1.48 m in each group with no significant difference, meaning the variety of elevation extracted from GE and actual elevation based on DEM was stable, and the GE elevation can reflect and present the complex terrain of research regions. Meanwhile, by analyzing DEM elevation of samples, there was no significant difference statistically between the elevation of samples for establishing CRM and that for validating CRM, which indicated that the sample grouping elevation data were suitable for the establishment and validation of the elevation correction model in this study.

Further compared GE elevation with DEM elevation in the Poyang Lake region, MAE and RMSE between them was 1.28 m and 3.95 m, respectively. In another case, Wang YS *et al*.^15^ compared the elevation extracted by GE in American with the elevation from National Elevation Dataset of United States Geological Survey (USGS NED), and found that the MAE and RMSE between them were 1.32 m and 2.27 m, respectively, which is correlated with our study.

Linear regression is a widely used statistical analysis method that uses regression analysis to determine the quantitative relationship between two or more variables^39^. This study found that there was a significant correlation between GE elevation and DEM elevation with r value up to 0.917. Consequently, a correction regression equation was established by using these two variables (GE elevation and DEM elevation) with high fitting degree.

The method of Cross-validation was often used to evaluate the accuracy of a mathematical model^40–43^. i.e. the majority of collected samples were used to establish the model, and the remaining samples were used to validate the model. By comparing MAE, RMSE and IOA before and after correction of GE elevation with DEM elevation, we can see that MAE, RMSE and IOA of GE elevation were 1.28 m, 3.95 m and-0.40 before correction, respectively. After GE elevation values are corrected by elevation conversion model, the MAE and RMSE were reduced to 0.74 m and 1.3 m correspondingly, while the IOA was increased to 0.34, which indicated that the elevation accuracy was greatly improved. Therefore, the accuracy of GE elevation can be improved by using the elevation conversion model, which can reflect and present the geomorphology precisely in practical application.

## Conclusion

In this study, we use limited observational data to correct the elevation values extracted through GE, aiming to find an acceptable, simple, effective and low-cost correction simulation method. The results indicated that the method can accurately located the extraction elevation. In general, the high-precision corrected elevation data obtained from GE can provide a reliable data source for various applications.

However, it should be noticed that GE only records elevation information on the ground, and if involved some buildings, for example, overpasses and tunnels, Google Earth elevation data may not be accurate. To deal with these abnormal elevation values, we can develop corresponding programs, such as using the elevation values of the surrounding non-obstacles to interpolate, or use the method of Ensemble Empirical Mode Decomposition (EEMD) to smooth the abnormal elevation^44^.

Generally, GE presents indisputable advantages such as widely used, free charge and rapid update, and it can directly or indirectly access to massive information related to topography, geomorphology and ground objects. However, it still needs to be converted into more reliable data by combining with pre-processing tools. Up to date, elevation data of snail-inhabited environment in epidemic areas with schistosomiasis is not available globally. In order to conduct researches related to elevation, it is suggested that GE can be used to extract the corresponding elevation and then improve the elevation value through establishing the elevation correction model, which will help to improve the reliability of related researches.

## Data Availability

The datasets generated and analyzed during the current study are available from the corresponding author on reasonable request.

## Code availability

The code generated during the current study are available from the corresponding author on reasonable request.
